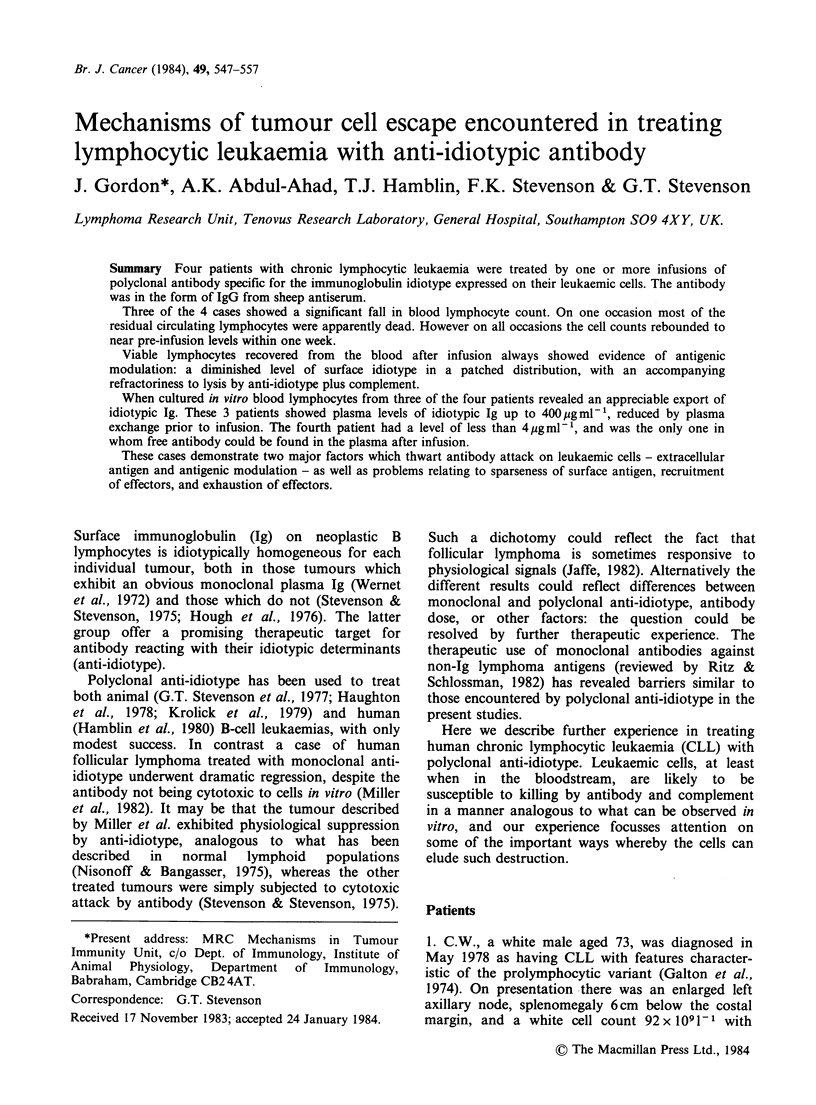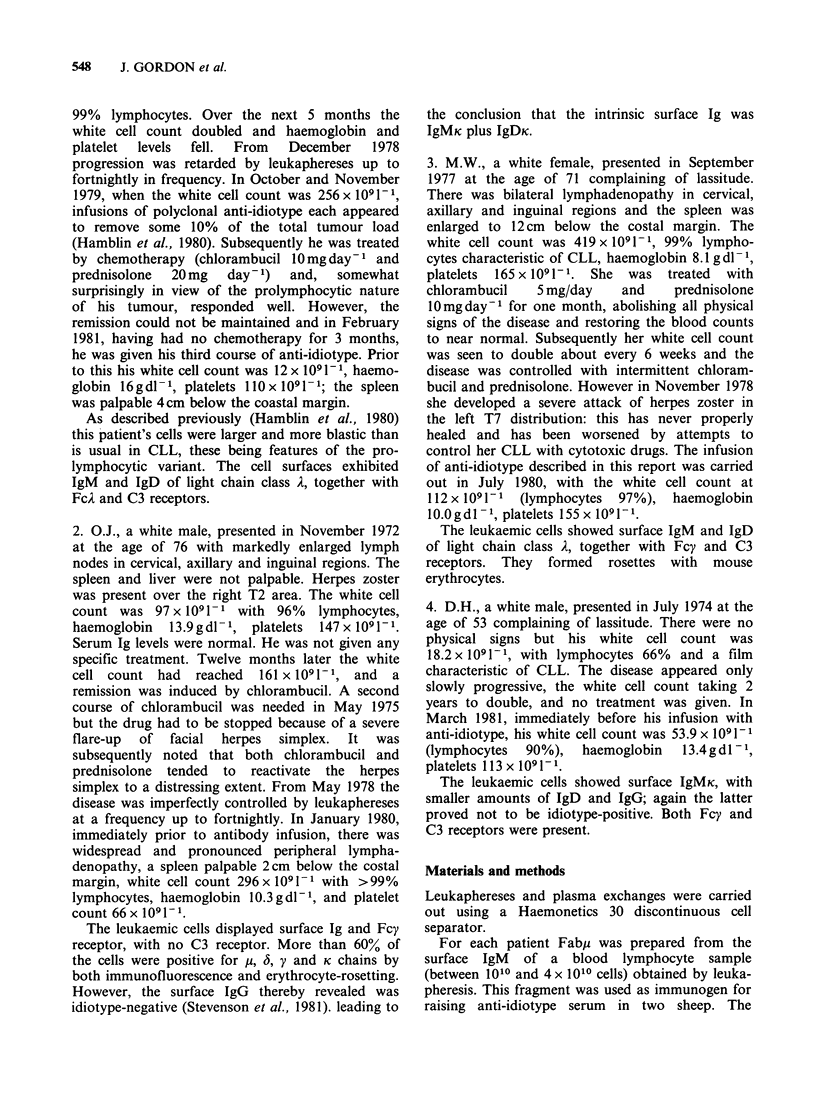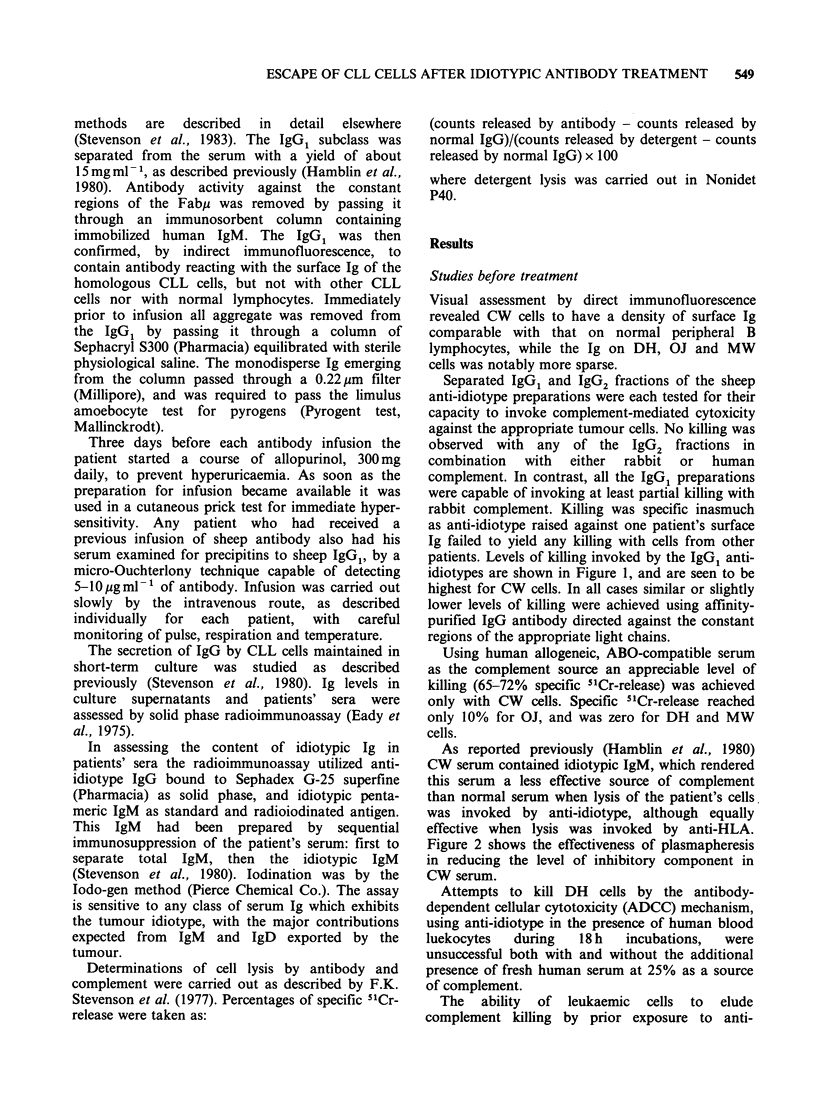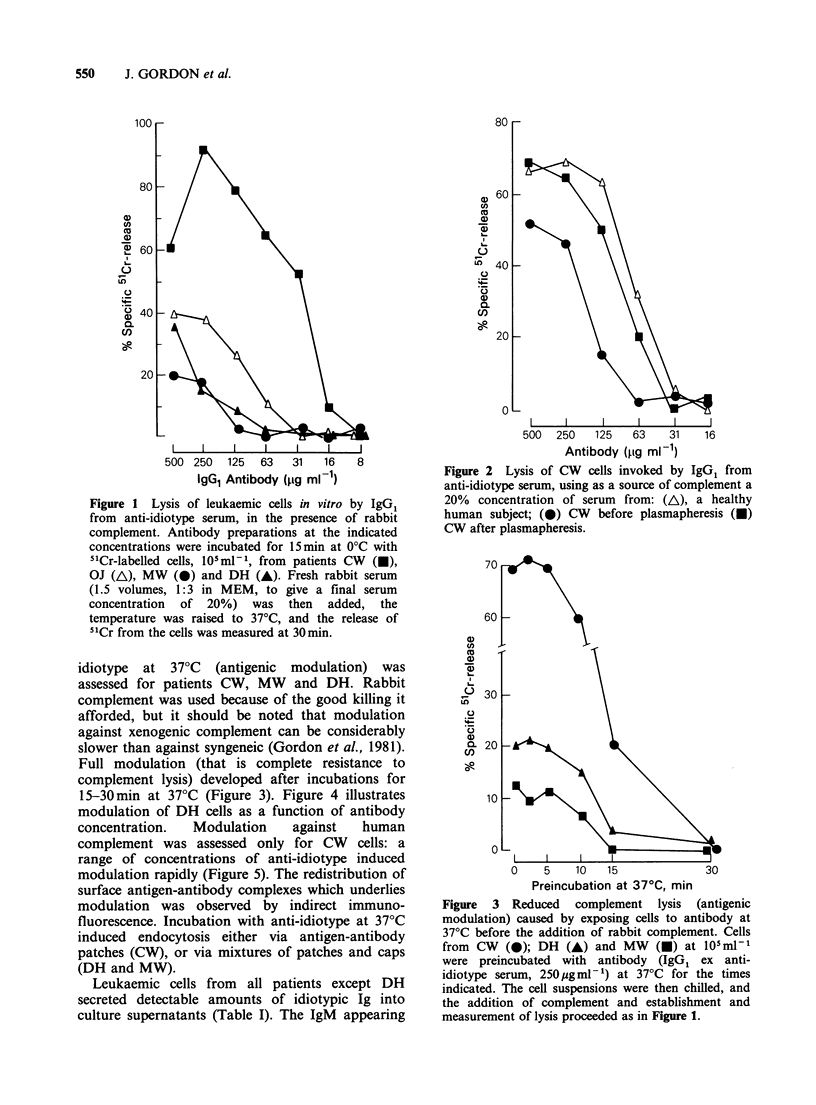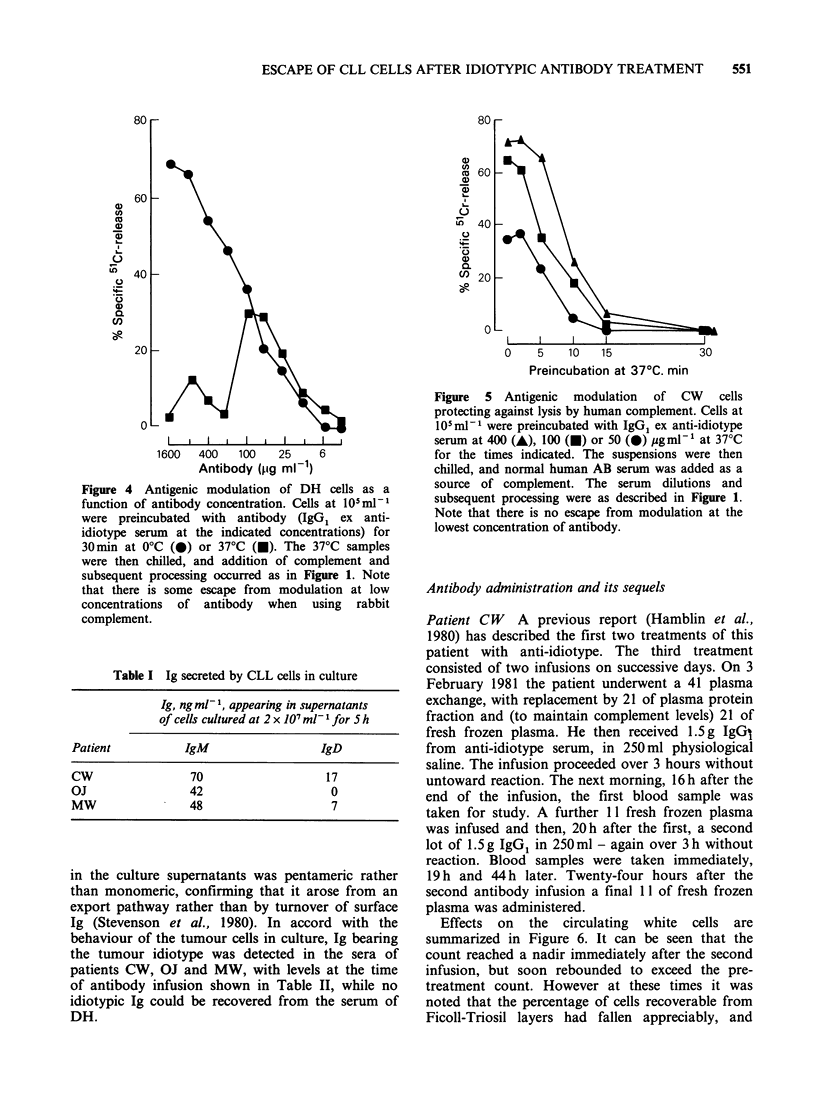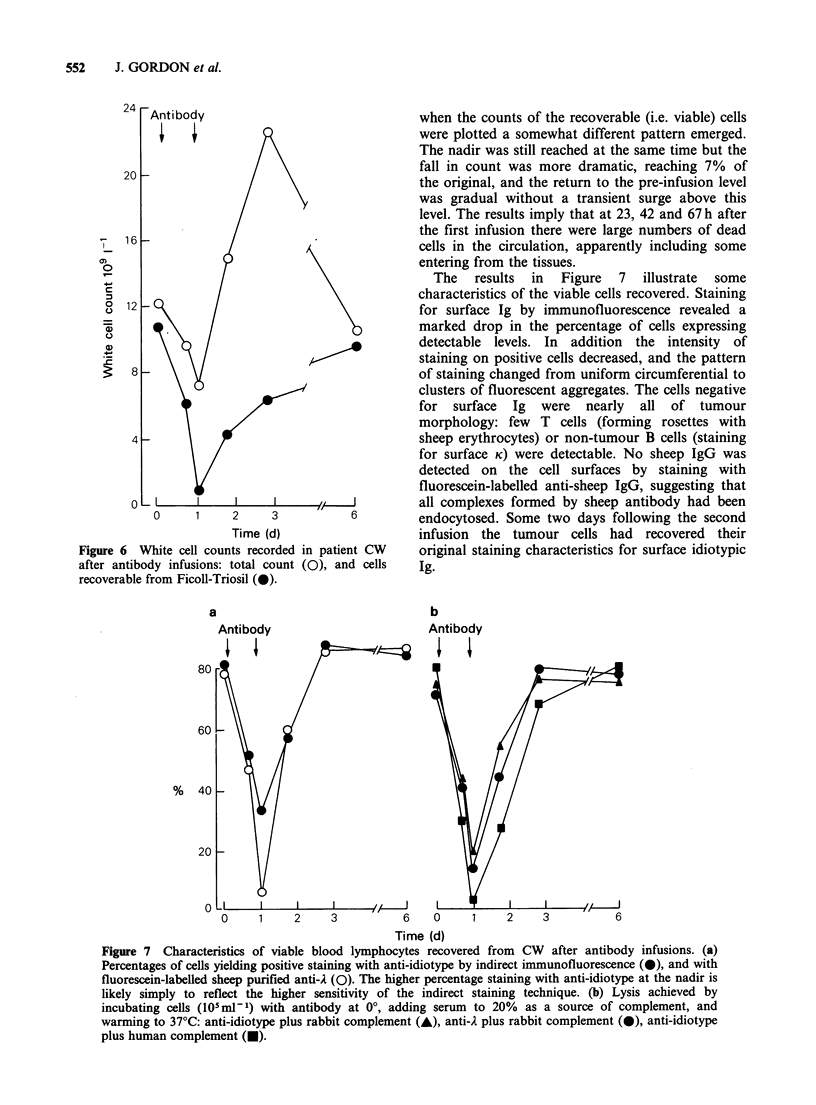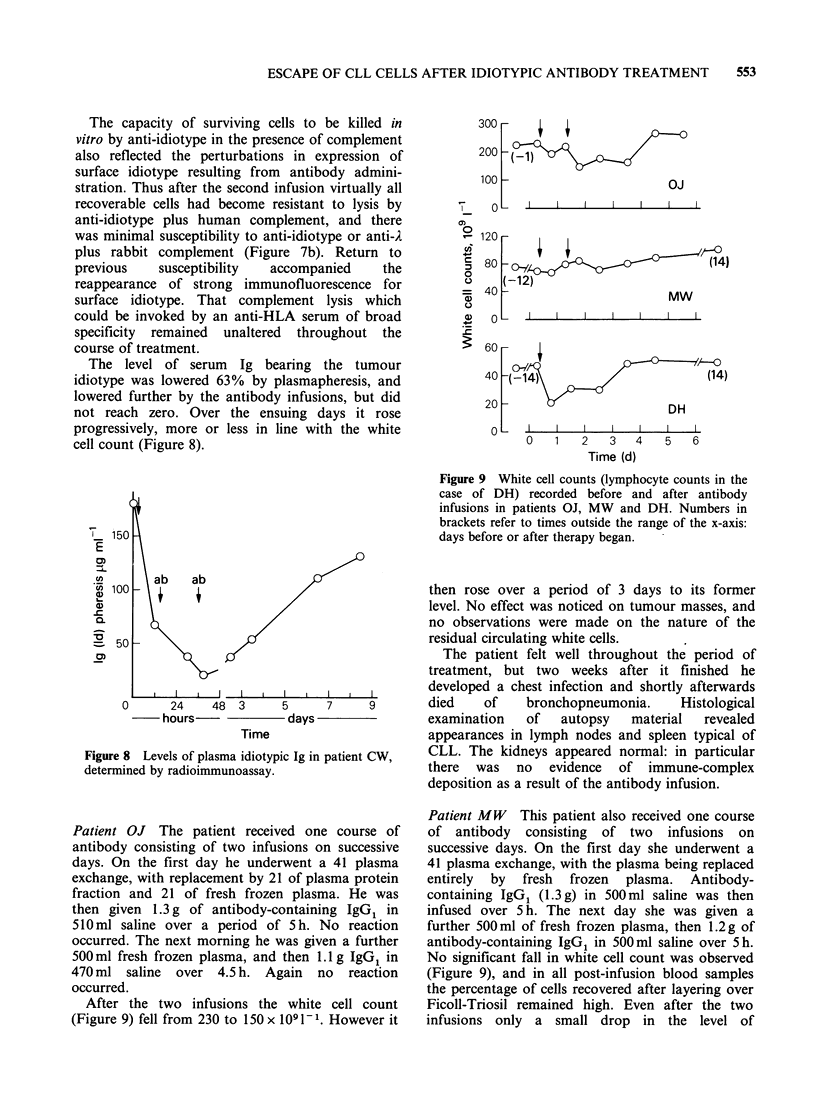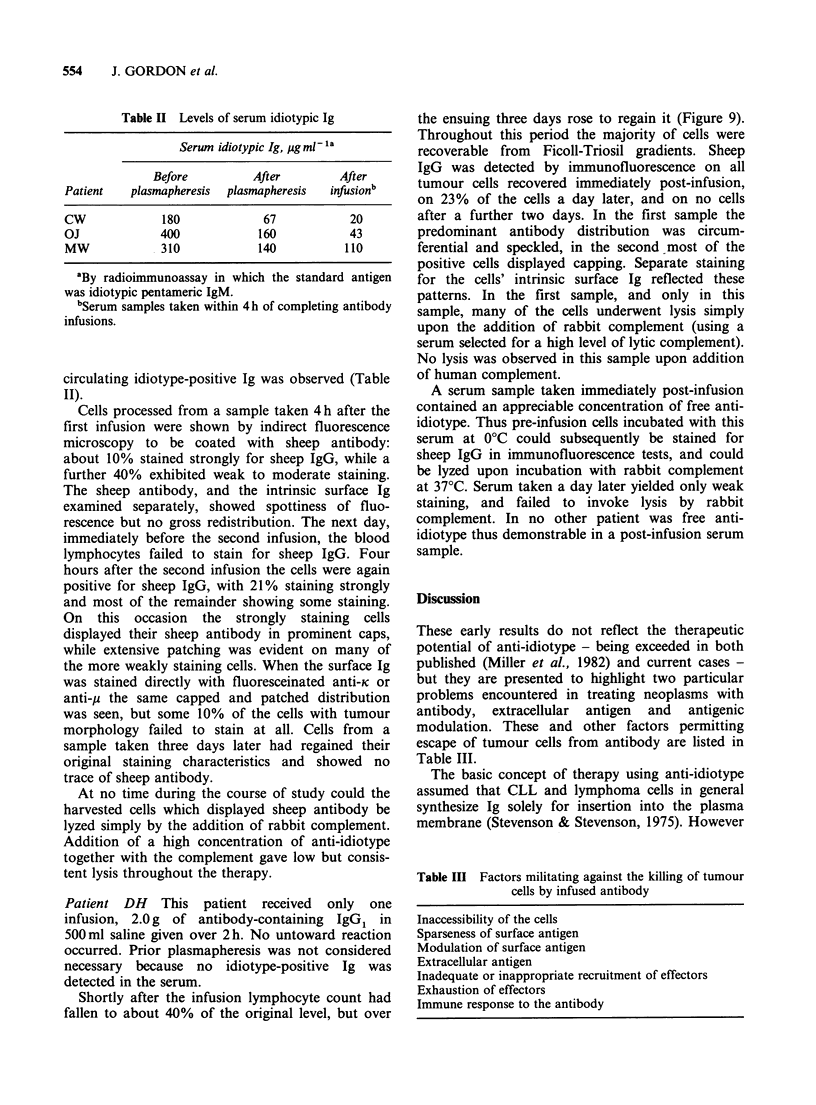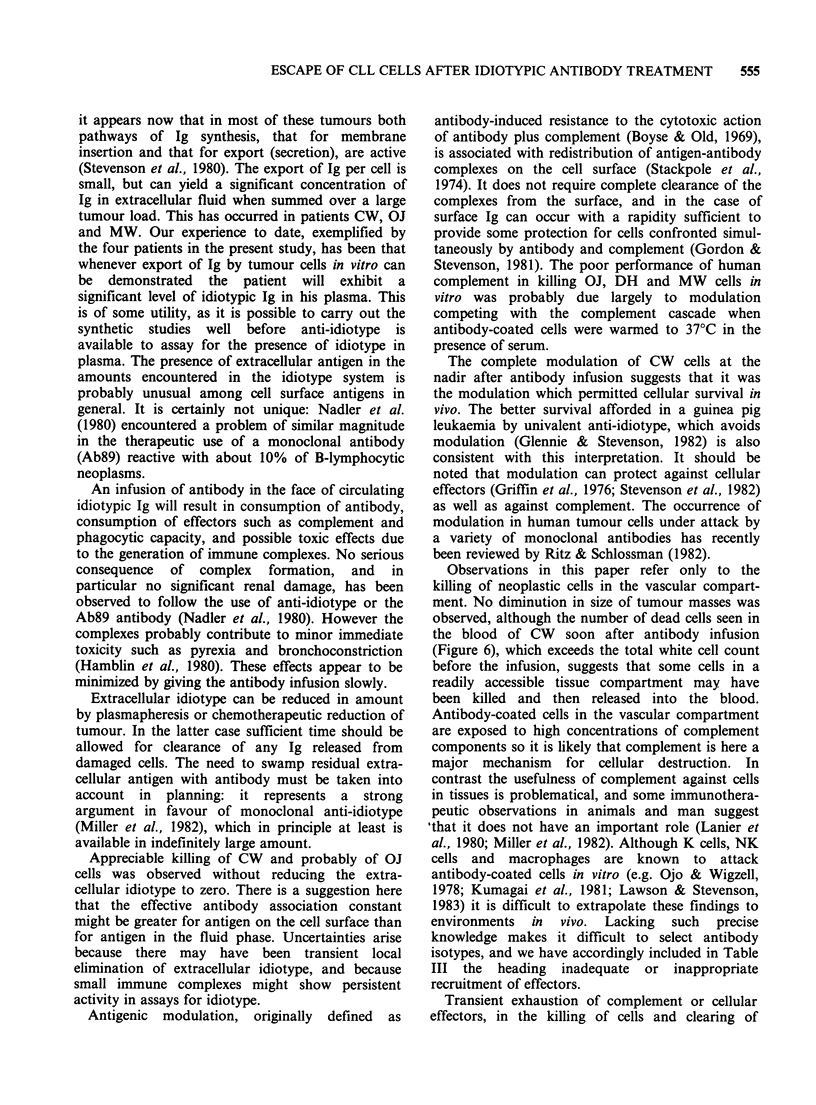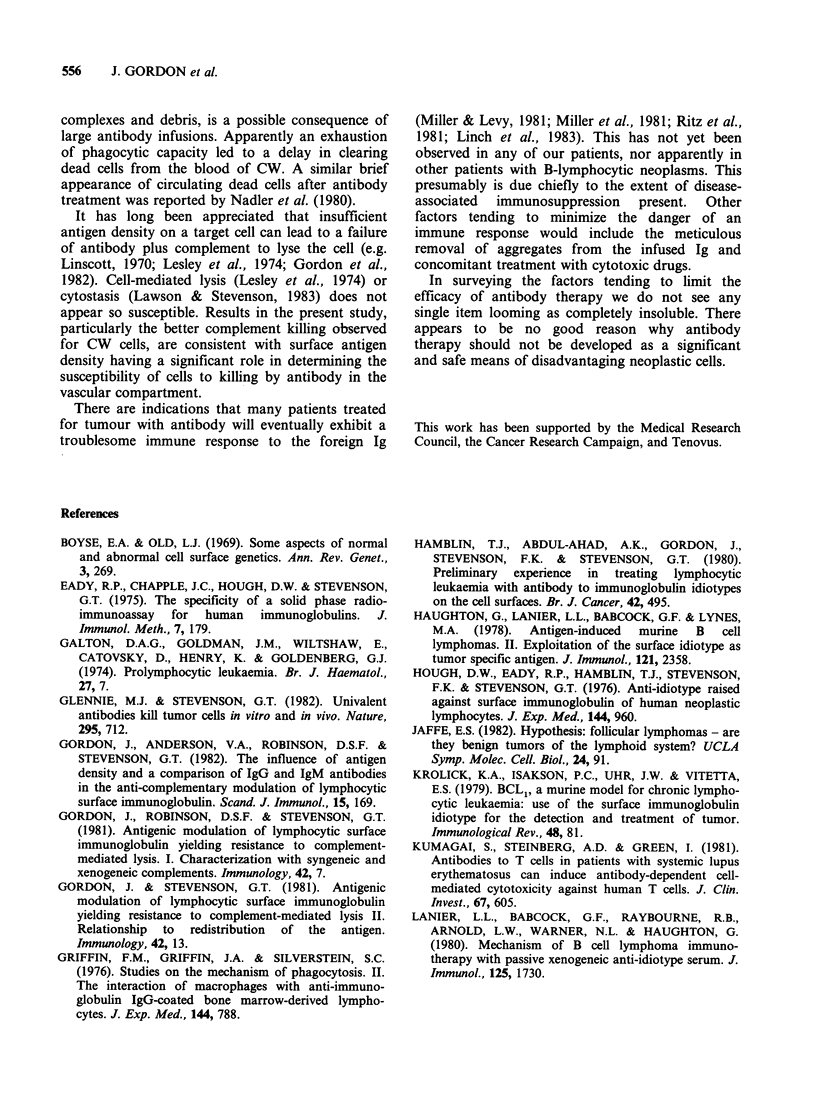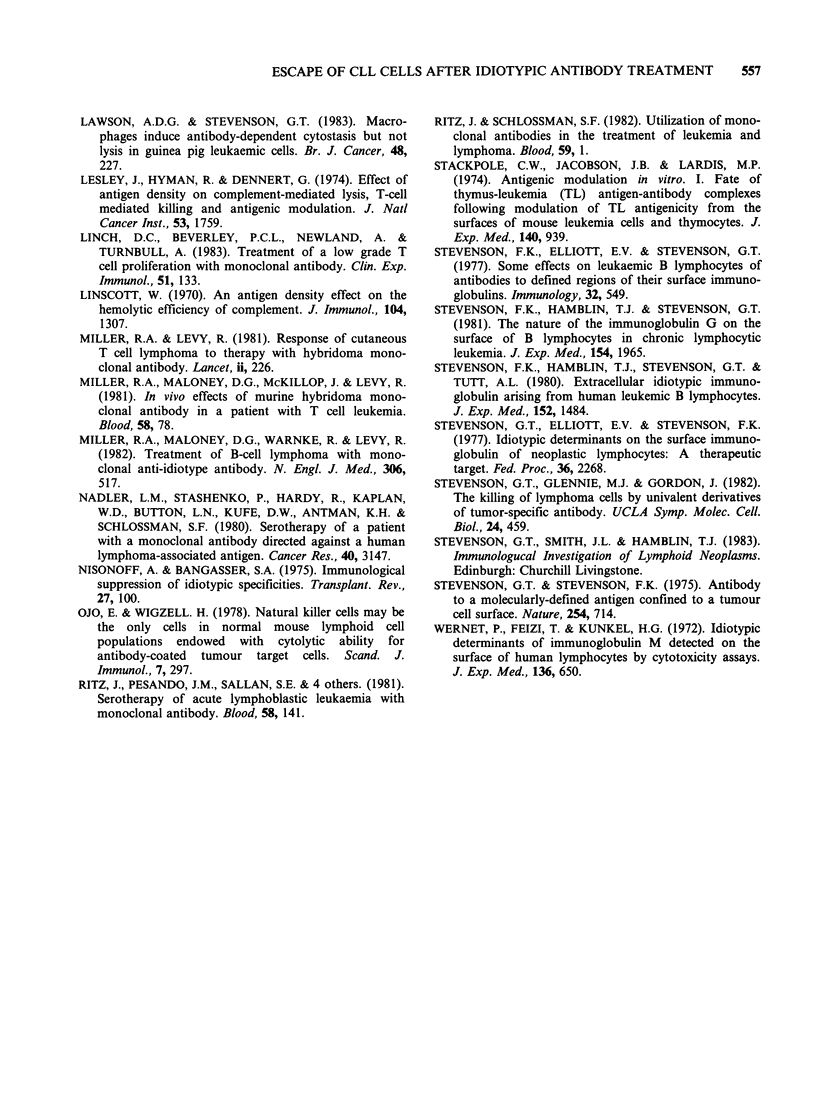# Mechanisms of tumour cell escape encountered in treating lymphocytic leukaemia with anti-idiotypic antibody.

**DOI:** 10.1038/bjc.1984.88

**Published:** 1984-05

**Authors:** J. Gordon, A. K. Abdul-Ahad, T. J. Hamblin, F. K. Stevenson, G. T. Stevenson

## Abstract

Four patients with chronic lymphocytic leukaemia were treated by one or more infusions of polyclonal antibody specific for the immunoglobulin idiotype expressed on their leukaemic cells. The antibody was in the form of IgG from sheep antiserum. Three of the 4 cases showed a significant fall in blood lymphocyte count. On one occasion most of the residual circulating lymphocytes were apparently dead. However on all occasions the cell counts rebounded to near pre-infusion levels within one week. Viable lymphocytes recovered from the blood after infusion always showed evidence of antigenic modulation: a diminished level of surface idiotype in a patched distribution, with an accompanying refractoriness to lysis by anti-idiotype plus complement. When cultured in vitro blood lymphocytes from three of the four patients revealed an appreciable export of idiotypic Ig. These 3 patients showed plasma levels of idiotypic Ig up to 400 micrograms ml-1, reduced by plasma exchange prior to infusion. The fourth patient had a level of less than 4 micrograms ml-1, and was the only one in whom free antibody could be found in the plasma after infusion. These cases demonstrate two major factors which thwart antibody attack on leukaemic cells--extracellular antigen and antigenic modulation--as well as problems relating to sparseness of surface antigen, recruitment of effectors, and exhaustion of effectors.


*Br. J. Cancer* (1984), **49**, 547–557

### Mechanisms of tumour cell escape encountered in treating lymphocytic leukaemia with anti-idiotypic antibody

J. Gordon*, A.K. Abdul-Ahad, T.J. Hamblin, F.K. Stevenson & G.T. Stevenson

*Lymphoma Research Unit, Tenovus Research Laboratory, General Hospital, Southampton SO9 4XY, UK.*

**Summary** Four patients with chronic lymphocytic leukaemia were treated by one or more infusions of polyclonal antibody specific for the immunoglobulin idiotype expressed on their leukaemic cells. The antibody was in the form of IgG from sheep antiserum.

Three of the 4 cases showed a significant fall in blood lymphocyte count. On one occasion most of the residual circulating lymphocytes were apparently dead. However on all occasions the cell counts rebounded to near pre-infusion levels within one week.

Viable lymphocytes recovered from the blood after infusion always showed evidence of antigenic modulation: a diminished level of surface idiotype in a patched distribution, with an accompanying refractoriness to lysis by anti-idiotype plus complement.

When cultured *in vitro* blood lymphocytes from three of the four patients revealed an appreciable export of idiotypic Ig. These 3 patients showed plasma levels of idiotypic Ig up to $400\,\mu g\,ml^{-1}$, reduced by plasma exchange prior to infusion. The fourth patient had a level of less than $4\,\mu g\,ml^{-1}$, and was the only one in whom free antibody could be found in the plasma after infusion.

These cases demonstrate two major factors which thwart antibody attack on leukaemic cells – extracellular antigen and antigenic modulation – as well as problems relating to sparseness of surface antigen, recruitment of effectors, and exhaustion of effectors.

Surface immunoglobulin (Ig) on neoplastic B lymphocytes is idiotypically homogeneous for each individual tumour, both in those tumours which exhibit an obvious monoclonal plasma Ig (Wernet *et al.*, 1972) and those which do not (Stevenson & Stevenson, 1975; Hough *et al.*, 1976). The latter group offer a promising therapeutic target for antibody reacting with their idiotypic determinants (anti-idiotype).

Polyclonal anti-idiotype has been used to treat both animal (G.T. Stevenson *et al.*, 1977; Haughton *et al.*, 1978; Krolick *et al.*, 1979) and human (Hamblin *et al.*, 1980) B-cell leukaemias, with only modest success. In contrast a case of human follicular lymphoma treated with monoclonal anti-idiotype underwent dramatic regression, despite the antibody not being cytotoxic to cells *in vitro* (Miller *et al.*, 1982). It may be that the tumour described by Miller *et al.* exhibited physiological suppression by anti-idiotype, analogous to what has been described in normal lymphoid populations (Nisonoff & Bangasser, 1975), whereas the other treated tumours were simply subjected to cytotoxic attack by antibody (Stevenson & Stevenson, 1975).

*Present address: MRC Mechanisms in Tumour Immunity Unit, c/o Dept. of Immunology, Institute of Animal Physiology, Department of Immunology, Babraham, Cambridge CB2 4AT.

Correspondence: G.T. Stevenson

Received 17 November 1983; accepted 24 January 1984.

Such a dichotomy could reflect the fact that follicular lymphoma is sometimes responsive to physiological signals (Jaffe, 1982). Alternatively the different results could reflect differences between monoclonal and polyclonal anti-idiotype, antibody dose, or other factors: the question could be resolved by further therapeutic experience. The therapeutic use of monoclonal antibodies against non-Ig lymphoma antigens (reviewed by Ritz & Schlossman, 1982) has revealed barriers similar to those encountered by polyclonal anti-idiotype in the present studies.

Here we describe further experience in treating human chronic lymphocytic leukaemia (CLL) with polyclonal anti-idiotype. Leukaemic cells, at least when in the bloodstream, are likely to be susceptible to killing by antibody and complement in a manner analogous to what can be observed *in vitro*, and our experience focusses attention on some of the important ways whereby the cells can elude such destruction.

### Patients

1. C.W., a white male aged 73, was diagnosed in May 1978 as having CLL with features characteristic of the prolymphocytic variant (Galton *et al.*, 1974). On presentation there was an enlarged left axillary node, splenomegaly 6 cm below the costal margin, and a white cell count $92\times10^{9}\,l^{-1}$ with

© The Macmillan Press Ltd., 1984

99% lymphocytes. Over the next 5 months the white cell count doubled and haemoglobin and platelet levels fell. From December 1978 progression was retarded by leukaphereses up to fortnightly in frequency. In October and November 1979, when the white cell count was $256 \times 10^9 l^{-1}$, infusions of polyclonal anti-idiotype each appeared to remove some 10% of the total tumour load (Hamblin *et al.*, 1980). Subsequently he was treated by chemotherapy (chlorambucil 10 mg day$^{-1}$ and prednisolone 20 mg day$^{-1}$) and, somewhat surprisingly in view of the prolymphocytic nature of his tumour, responded well. However, the remission could not be maintained and in February 1981, having had no chemotherapy for 3 months, he was given his third course of anti-idiotype. Prior to this his white cell count was $12 \times 10^9 l^{-1}$, haemoglobin 16 g dl$^{-1}$, platelets $110 \times 10^9 l^{-1}$; the spleen was palpable 4 cm below the coastal margin.

As described previously (Hamblin *et al.*, 1980) this patient's cells were larger and more blastic than is usual in CLL, these being features of the prolymphocytic variant. The cell surfaces exhibited IgM and IgD of light chain class $\lambda$, together with Fc$\lambda$ and C3 receptors.

2. O.J., a white male, presented in November 1972 at the age of 76 with markedly enlarged lymph nodes in cervical, axillary and inguinal regions. The spleen and liver were not palpable. Herpes zoster was present over the right T2 area. The white cell count was $97 \times 10^9 l^{-1}$ with 96% lymphocytes, haemoglobin 13.9 g dl$^{-1}$, platelets $147 \times 10^9 l^{-1}$. Serum Ig levels were normal. He was not given any specific treatment. Twelve months later the white cell count had reached $161 \times 10^9 l^{-1}$, and a remission was induced by chlorambucil. A second course of chlorambucil was needed in May 1975 but the drug had to be stopped because of a severe flare-up of facial herpes simplex. It was subsequently noted that both chlorambucil and prednisolone tended to reactivate the herpes simplex to a distressing extent. From May 1978 the disease was imperfectly controlled by leukaphereses at a frequency up to fortnightly. In January 1980, immediately prior to antibody infusion, there was widespread and pronounced peripheral lymphadenopathy, a spleen palpable 2 cm below the costal margin, white cell count $296 \times 10^9 l^{-1}$ with >99% lymphocytes, haemoglobin 10.3 g dl$^{-1}$, and platelet count $66 \times 10^9 l^{-1}$.

The leukaemic cells displayed surface Ig and Fc$\gamma$ receptor, with no C3 receptor. More than 60% of the cells were positive for $\mu$, $\delta$, $\gamma$ and $\kappa$ chains by both immunofluorescence and erythrocyte-rosetting. However, the surface IgG thereby revealed was idiotype-negative (Stevenson *et al.*, 1981). leading to the conclusion that the intrinsic surface Ig was IgM$\kappa$ plus IgD$\kappa$.

3. M.W., a white female, presented in September 1977 at the age of 71 complaining of lassitude. There was bilateral lymphadenopathy in cervical, axillary and inguinal regions and the spleen was enlarged to 12 cm below the costal margin. The white cell count was $419 \times 10^9 l^{-1}$, 99% lymphocytes characteristic of CLL, haemoglobin 8.1 g dl$^{-1}$, platelets $165 \times 10^9 l^{-1}$. She was treated with chlorambucil 5 mg/day and prednisolone 10 mg day$^{-1}$ for one month, abolishing all physical signs of the disease and restoring the blood counts to near normal. Subsequently her white cell count was seen to double about every 6 weeks and the disease was controlled with intermittent chlorambucil and prednisolone. However in November 1978 she developed a severe attack of herpes zoster in the left T7 distribution: this has never properly healed and has been worsened by attempts to control her CLL with cytotoxic drugs. The infusion of anti-idiotype described in this report was carried out in July 1980, with the white cell count at $112 \times 10^9 l^{-1}$ (lymphocytes 97%), haemoglobin 10.0 g dl$^{-1}$, platelets $155 \times 10^9 l^{-1}$.

The leukaemic cells showed surface IgM and IgD of light chain class $\lambda$, together with Fc$\gamma$ and C3 receptors. They formed rosettes with mouse erythrocytes.

4. D.H., a white male, presented in July 1974 at the age of 53 complaining of lassitude. There were no physical signs but his white cell count was $18.2 \times 10^9 l^{-1}$, with lymphocytes 66% and a film characteristic of CLL. The disease appeared only slowly progressive, the white cell count taking 2 years to double, and no treatment was given. In March 1981, immediately before his infusion with anti-idiotype, his white cell count was $53.9 \times 10^9 l^{-1}$ (lymphocytes 90%), haemoglobin 13.4 g dl$^{-1}$, platelets $113 \times 10^9 l^{-1}$.

The leukaemic cells showed surface IgM$\kappa$, with smaller amounts of IgD and IgG; again the latter proved not to be idiotype-positive. Both Fc$\gamma$ and C3 receptors were present.

### Materials and methods

Leukaphereses and plasma exchanges were carried out using a Haemonetics 30 discontinuous cell separator.

For each patient Fab$\mu$ was prepared from the surface IgM of a blood lymphocyte sample (between $10^{10}$ and $4 \times 10^{10}$ cells) obtained by leukapheresis. This fragment was used as immunogen for raising anti-idiotype serum in two sheep. The methods are described in detail elsewhere (Stevenson *et al.*, 1983). The $IgG_1$ subclass was separated from the serum with a yield of about $15 mg ml^{-1}$, as described previously (Hamblin *et al.*, 1980). Antibody activity against the constant regions of the Fab$\mu$ was removed by passing it through an immunosorbent column containing immobilized human IgM. The $IgG_1$ was then confirmed, by indirect immunofluorescence, to contain antibody reacting with the surface Ig of the homologous CLL cells, but not with other CLL cells nor with normal lymphocytes. Immediately prior to infusion all aggregate was removed from the $IgG_1$ by passing it through a column of Sephacryl S300 (Pharmacia) equilibrated with sterile physiological saline. The monodisperse Ig emerging from the column passed through a $0.22 \mu m$ filter (Millipore), and was required to pass the limulus amoebocyte test for pyrogens (Pyrogent test, Mallinckrodt).

Three days before each antibody infusion the patient started a course of allopurinol, 300 mg daily, to prevent hyperuricaemia. As soon as the preparation for infusion became available it was used in a cutaneous prick test for immediate hypersensitivity. Any patient who had received a previous infusion of sheep antibody also had his serum examined for precipitins to sheep $IgG_1$, by a micro-Ouchterlony technique capable of detecting $5–10 \mu g ml^{-1}$ of antibody. Infusion was carried out slowly by the intravenous route, as described individually for each patient, with careful monitoring of pulse, respiration and temperature.

The secretion of IgG by CLL cells maintained in short-term culture was studied as described previously (Stevenson *et al.*, 1980). Ig levels in culture supernatants and patients' sera were assessed by solid phase radioimmunoassay (Eady *et al.*, 1975).

In assessing the content of idiotypic Ig in patients' sera the radioimmunoassay utilized anti-idiotype IgG bound to Sephadex G-25 superfine (Pharmacia) as solid phase, and idiotypic pentameric IgM as standard and radioiodinated antigen. This IgM had been prepared by sequential immunosuppression of the patient's serum: first to separate total IgM, then the idiotypic IgM (Stevenson *et al.*, 1980). Iodination was by the Iodo-gen method (Pierce Chemical Co.). The assay is sensitive to any class of serum Ig which exhibits the tumour idiotype, with the major contributions expected from IgM and IgD exported by the tumour.

Determinations of cell lysis by antibody and complement were carried out as described by F.K. Stevenson *et al.* (1977). Percentages of specific $^{51}Cr$-release were taken as:

(counts released by antibody – counts released by normal IgG)/(counts released by detergent – counts released by normal IgG) $\times$ 100

where detergent lysis was carried out in Nonidet P40.

### Results

### *Studies before treatment*

Visual assessment by direct immunofluorescence revealed CW cells to have a density of surface Ig comparable with that on normal peripheral B lymphocytes, while the Ig on DH, OJ and MW cells was notably more sparse.

Separated $IgG_1$ and $IgG_2$ fractions of the sheep anti-idiotype preparations were each tested for their capacity to invoke complement-mediated cytoxicity against the appropriate tumour cells. No killing was observed with any of the $IgG_2$ fractions in combination with either rabbit or human complement. In contrast, all the $IgG_1$ preparations were capable of invoking at least partial killing with rabbit complement. Killing was specific inasmuch as anti-idiotype raised against one patient's surface Ig failed to yield any killing with cells from other patients. Levels of killing invoked by the $IgG_1$ anti-idiotypes are shown in Figure 1, and are seen to be highest for CW cells. In all cases similar or slightly lower levels of killing were achieved using affinity-purified IgG antibody directed against the constant regions of the appropriate light chains.

Using human allogeneic, ABO-compatible serum as the complement source an appreciable level of killing (65–72% specific $^{51}Cr$-release) was achieved only with CW cells. Specific $^{51}Cr$-release reached only 10% for OJ, and was zero for DH and MW cells.

As reported previously (Hamblin *et al.*, 1980) CW serum contained idiotypic IgM, which rendered this serum a less effective source of complement than normal serum when lysis of the patient's cells was invoked by anti-idiotype, although equally effective when lysis was invoked by anti-HLA. Figure 2 shows the effectiveness of plasmapheresis in reducing the level of inhibitory component in CW serum.

Attempts to kill DH cells by the antibody-dependent cellular cytotoxicity (ADCC) mechanism, using anti-idiotype in the presence of human blood luekocytes during 18 h incubations, were unsuccessful both with and without the additional presence of fresh human serum at 25% as a source of complement.

The ability of leukaemic cells to elude complement killing by prior exposure to anti-

**Figure 1** Lysis of leukaemic cells *in vitro* by $IgG_1$ from anti-idiotype serum, in the presence of rabbit complement. Antibody preparations at the indicated concentrations were incubated for 15 min at 0°C with $^{51}Cr$-labelled cells, $10^5 ml^{-1}$, from patients CW (■), OJ (△), MW (●) and DH (▲). Fresh rabbit serum (1.5 volumes, 1:3 in MEM, to give a final serum concentration of 20%) was then added, the temperature was raised to 37°C, and the release of $^{51}Cr$ from the cells was measured at 30 min.

**Figure 2** Lysis of CW cells invoked by $IgG_1$ from anti-idiotype serum, using as a source of complement a 20% concentration of serum from: (△), a healthy human subject; (●) CW before plasmapheresis (■) CW after plasmapheresis.

idiotype at 37°C (antigenic modulation) was assessed for patients CW, MW and DH. Rabbit complement was used because of the good killing it afforded, but it should be noted that modulation against xenogenic complement can be considerably slower than against syngeneic (Gordon *et al.*, 1981). Full modulation (that is complete resistance to complement lysis) developed after incubations for 15–30 min at 37°C (Figure 3). Figure 4 illustrates modulation of DH cells as a function of antibody concentration. Modulation against human complement was assessed only for CW cells: a range of concentrations of anti-idiotype induced modulation rapidly (Figure 5). The redistribution of surface antigen-antibody complexes which underlies modulation was observed by indirect immunofluorescence. Incubation with anti-idiotype at 37°C induced endocytosis either via antigen-antibody patches (CW), or via mixtures of patches and caps (DH and MW).

Leukaemic cells from all patients except DH secreted detectable amounts of idiotypic Ig into culture supernatants (Table I). The IgM appearing

**Figure 3** Reduced complement lysis (antigenic modulation) caused by exposing cells to antibody at 37°C before the addition of rabbit complement. Cells from CW (●); DH (▲) and MW (■) at $10^5 ml^{-1}$ were preincubated with antibody ($IgG_1$ ex anti-idiotype serum, $250 \mu g ml^{-1}$) at 37°C for the times indicated. The cell suspensions were then chilled, and the addition of complement and establishment and measurement of lysis proceeded as in **Figure 1**.

**Figure 4** Antigenic modulation of DH cells as a function of antibody concentration. Cells at $10^5 ml^{-1}$ were preincubated with antibody ($IgG_1$ ex anti-idiotype serum at the indicated concentrations) for 30 min at 0°C (●) or 37°C (■). The 37°C samples were then chilled, and addition of complement and subsequent processing occurred as in **Figure 1**. Note that there is some escape from modulation at low concentrations of antibody when using rabbit complement.

**Table I** Ig secreted by CLL cells in culture

| | *Ig, ng ml$^{-1}$, appearing in supernatants of cells cultured at 2 × 10$^7$ ml$^{-1}$ for 5 h* | |
|---|---|---|
| *Patient* | *IgM* | *IgD* |
| CW | 70 | 17 |
| OJ | 42 | 0 |
| MW | 48 | 7 |

in the culture supernatants was pentameric rather than monomeric, confirming that it arose from an export pathway rather than by turnover of surface Ig (Stevenson *et al.*, 1980). In accord with the behaviour of the tumour cells in culture, Ig bearing the tumour idiotype was detected in the sera of patients CW, OJ and MW, with levels at the time of antibody infusion shown in Table II, while no idiotypic Ig could be recovered from the serum of DH.

**Figure 5** Antigenic modulation of CW cells protecting against lysis by human complement. Cells at $10^5 ml^{-1}$ were preincubated with $IgG_1$ ex anti-idiotype serum at 400 (▲), 100 (■) or 50 (●) $\mu g ml^{-1}$ at 37°C for the times indicated. The suspensions were then chilled, and normal human AB serum was added as a source of complement. The serum dilutions and subsequent processing were as described in **Figure 1**. Note that there is no escape from modulation at the lowest concentration of antibody.

### *Antibody administration and its sequels*

*Patient CW* A previous report (Hamblin *et al.*, 1980) has described the first two treatments of this patient with anti-idiotype. The third treatment consisted of two infusions on successive days. On 3 February 1981 the patient underwent a 41 plasma exchange, with replacement by 21 of plasma protein fraction and (to maintain complement levels) 21 of fresh frozen plasma. He then received 1.5 g $IgG_1$ from anti-idiotype serum, in 250 ml physiological saline. The infusion proceeded over 3 hours without untoward reaction. The next morning, 16 h after the end of the infusion, the first blood sample was taken for study. A further 11 fresh frozen plasma was infused and then, 20 h after the first, a second lot of 1.5 g $IgG_1$ in 250 ml – again over 3 h without reaction. Blood samples were taken immediately, 19 h and 44 h later. Twenty-four hours after the second antibody infusion a final 11 of fresh frozen plasma was administered.

Effects on the circulating white cells are summarized in Figure 6. It can be seen that the count reached a nadir immediately after the second infusion, but soon rebounded to exceed the pre-treatment count. However at these times it was noted that the percentage of cells recoverable from Ficoll-Triosil layers had fallen appreciably, and

**Figure 6** White cell counts recorded in patient CW after antibody infusions: total count (○), and cells recoverable from Ficoll-Triosil (●).

when the counts of the recoverable (i.e. viable) cells were plotted a somewhat different pattern emerged. The nadir was still reached at the same time but the fall in count was more dramatic, reaching 7% of the original, and the return to the pre-infusion level was gradual without a transient surge above this level. The results imply that at 23, 42 and 67 h after the first infusion there were large numbers of dead cells in the circulation, apparently including some entering from the tissues.

The results in Figure 7 illustrate some characteristics of the viable cells recovered. Staining for surface Ig by immunofluorescence revealed a marked drop in the percentage of cells expressing detectable levels. In addition the intensity of staining on positive cells decreased, and the pattern of staining changed from uniform circumferential to clusters of fluorescent aggregates. The cells negative for surface Ig were nearly all of tumour morphology: few T cells (forming rosettes with sheep erythrocytes) or non-tumour B cells (staining for surface $\kappa$) were detectable. No sheep IgG was detected on the cell surfaces by staining with fluorescein-labelled anti-sheep IgG, suggesting that all complexes formed by sheep antibody had been endocytosed. Some two days following the second infusion the tumour cells had recovered their original staining characteristics for surface idiotypic Ig.

**Figure 7** Characteristics of viable blood lymphocytes recovered from CW after antibody infusions. (**a**) Percentages of cells yielding positive staining with anti-idiotype by indirect immunofluorescence (●), and with fluorescein-labelled sheep purified anti-$\lambda$ (○). The higher percentage staining with anti-idiotype at the nadir is likely simply to reflect the higher sensitivity of the indirect staining technique. (**b**) Lysis achieved by incubating cells ($10^5$ ml$^{-1}$) with antibody at 0°, adding serum to 20% as a source of complement, and warming to 37°C: anti-idiotype plus rabbit complement (▲), anti-$\lambda$ plus rabbit complement (●), anti-idiotype plus human complement (■).

The capacity of surviving cells to be killed *in vitro* by anti-idiotype in the presence of complement also reflected the perturbations in expression of surface idiotype resulting from antibody administration. Thus after the second infusion virtually all recoverable cells had become resistant to lysis by anti-idiotype plus human complement, and there was minimal susceptibility to anti-idiotype or anti-$\lambda$ plus rabbit complement (Figure 7b). Return to previous susceptibility accompanied the reappearance of strong immunofluorescence for surface idiotype. That complement lysis which could be invoked by an anti-HLA serum of broad specificity remained unaltered throughout the course of treatment.

The level of serum Ig bearing the tumour idiotype was lowered 63% by plasmapheresis, and lowered further by the antibody infusions, but did not reach zero. Over the ensuing days it rose progressively, more or less in line with the white cell count (Figure 8).

**Figure 8** Levels of plasma idiotypic Ig in patient CW, determined by radioimmunoassay.

**Figure 9** White cell counts (lymphocyte counts in the case of DH) recorded before and after antibody infusions in patients OJ, MW and DH. Numbers in brackets refer to times outside the range of the x-axis: days before or after therapy began.

*Patient OJ* The patient received one course of antibody consisting of two infusions on successive days. On the first day he underwent a 41 plasma exchange, with replacement by 21 of plasma protein fraction and 21 of fresh frozen plasma. He was then given 1.3 g of antibody-containing $IgG_1$ in 510 ml saline over a period of 5 h. No reaction occurred. The next morning he was given a further 500 ml fresh frozen plasma, and then 1.1 g $IgG_1$ in 470 ml saline over 4.5 h. Again no reaction occurred.

After the two infusions the white cell count (Figure 9) fell from 230 to $150 \times 10^9 l^{-1}$. However it then rose over a period of 3 days to its former level. No effect was noticed on tumour masses, and no observations were made on the nature of the residual circulating white cells.

The patient felt well throughout the period of treatment, but two weeks after it finished he developed a chest infection and shortly afterwards died of bronchopneumonia. Histological examination of autopsy material revealed appearances in lymph nodes and spleen typical of CLL. The kidneys appeared normal: in particular there was no evidence of immune-complex deposition as a result of the antibody infusion.

*Patient MW* This patient also received one course of antibody consisting of two infusions on successive days. On the first day she underwent a 41 plasma exchange, with the plasma being replaced entirely by fresh frozen plasma. Antibody-containing $IgG_1$ (1.3 g) in 500 ml saline was then infused over 5 h. The next day she was given a further 500 ml of fresh frozen plasma, then 1.2 g of antibody-containing $IgG_1$ in 500 ml saline over 5 h. No significant fall in white cell count was observed (Figure 9), and in all post-infusion blood samples the percentage of cells recovered after layering over Ficoll-Triosil remained high. Even after the two infusions only a small drop in the level of

**Table II** Levels of serum idiotypic Ig

| Patient | *Serum idiotypic Ig, μg ml$^{-1}$* [a] | | |
|---|---|---|---|
| | *Before plasmapheresis* | *After plasmapheresis* | *After infusion* [b] |
| CW | 180 | 67 | 20 |
| OJ | 400 | 160 | 43 |
| MW | 310 | 140 | 110 |

[a]By radioimmunoassay in which the standard antigen was idiotypic pentameric IgM.

[b]Serum samples taken within 4 h of completing antibody infusions.

circulating idiotype-positive Ig was observed (Table II).

Cells processed from a sample taken 4 h after the first infusion were shown by indirect fluorescence microscopy to be coated with sheep antibody: about 10% stained strongly for sheep IgG, while a further 40% exhibited weak to moderate staining. The sheep antibody, and the intrinsic surface Ig examined separately, showed spottiness of fluorescence but no gross redistribution. The next day, immediately before the second infusion, the blood lymphocytes failed to stain for sheep IgG. Four hours after the second infusion the cells were again positive for sheep IgG, with 21% staining strongly and most of the remainder showing some staining. On this occasion the strongly staining cells displayed their sheep antibody in prominent caps, while extensive patching was evident on many of the more weakly staining cells. When the surface Ig was stained directly with fluoresceinated anti-$\kappa$ or anti-$\mu$ the same capped and patched distribution was seen, but some 10% of the cells with tumour morphology failed to stain at all. Cells from a sample taken three days later had regained their original staining characteristics and showed no trace of sheep antibody.

At no time during the course of study could the harvested cells which displayed sheep antibody be lyzed simply by the addition of rabbit complement. Addition of a high concentration of anti-idiotype together with the complement gave low but consistent lysis throughout the therapy.

*Patient DH* This patient received only one infusion, 2.0 g of antibody-containing $IgG_1$ in 500 ml saline given over 2 h. No untoward reaction occurred. Prior plasmapheresis was not considered necessary because no idiotype-positive Ig was detected in the serum.

Shortly after the infusion lymphocyte count had fallen to about 40% of the original level, but over the ensuing three days rose to regain it (Figure 9). Throughout this period the majority of cells were recoverable from Ficoll-Triosil gradients. Sheep IgG was detected by immunofluorescence on all tumour cells recovered immediately post-infusion, on 23% of the cells a day later, and on no cells after a further two days. In the first sample the predominant antibody distribution was circumferential and speckled, in the second most of the positive cells displayed capping. Separate staining for the cells' intrinsic surface Ig reflected these patterns. In the first sample, and only in this sample, many of the cells underwent lysis simply upon the addition of rabbit complement (using a serum selected for a high level of lytic complement). No lysis was observed in this sample upon addition of human complement.

A serum sample taken immediately post-infusion contained an appreciable concentration of free anti-idiotype. Thus pre-infusion cells incubated with this serum at 0°C could subsequently be stained for sheep IgG in immunofluorescence tests, and could be lyzed upon incubation with rabbit complement at 37°C. Serum taken a day later yielded only weak staining, and failed to invoke lysis by rabbit complement. In no other patient was free anti-idiotype thus demonstrable in a post-infusion serum sample.

### Discussion

These early results do not reflect the therapeutic potential of anti-idiotype – being exceeded in both published (Miller *et al.*, 1982) and current cases – but they are presented to highlight two particular problems encountered in treating neoplasms with antibody, extracellular antigen and antigenic modulation. These and other factors permitting escape of tumour cells from antibody are listed in Table III.

The basic concept of therapy using anti-idiotype assumed that CLL and lymphoma cells in general synthesize Ig solely for insertion into the plasma membrane (Stevenson & Stevenson, 1975). However

**Table III** Factors militating against the killing of tumour cells by infused antibody

| |
|---|
| Inaccessibility of the cells |
| Sparseness of surface antigen |
| Modulation of surface antigen |
| Extracellular antigen |
| Inadequate or inappropriate recruitment of effectors |
| Exhaustion of effectors |
| Immune response to the antibody |

it appears now that in most of these tumours both pathways of Ig synthesis, that for membrane insertion and that for export (secretion), are active (Stevenson *et al.*, 1980). The export of Ig per cell is small, but can yield a significant concentration of Ig in extracellular fluid when summed over a large tumour load. This has occurred in patients CW, OJ and MW. Our experience to date, exemplified by the four patients in the present study, has been that whenever export of Ig by tumour cells *in vitro* can be demonstrated the patient will exhibit a significant level of idiotypic Ig in his plasma. This is of some utility, as it is possible to carry out the synthetic studies well before anti-idiotype is available to assay for the presence of idiotype in plasma. The presence of extracellular antigen in the amounts encountered in the idiotype system is probably unusual among cell surface antigens in general. It is certainly not unique: Nadler *et al.* (1980) encountered a problem of similar magnitude in the therapeutic use of a monoclonal antibody (Ab89) reactive with about 10% of B-lymphocytic neoplasms.

An infusion of antibody in the face of circulating idiotypic Ig will result in consumption of antibody, consumption of effectors such as complement and phagocytic capacity, and possible toxic effects due to the generation of immune complexes. No serious consequence of complex formation, and in particular no significant renal damage, has been observed to follow the use of anti-idiotype or the Ab89 antibody (Nadler *et al.*, 1980). However the complexes probably contribute to minor immediate toxicity such as pyrexia and bronchoconstriction (Hamblin *et al.*, 1980). These effects appear to be minimized by giving the antibody infusion slowly.

Extracellular idiotype can be reduced in amount by plasmapheresis or chemotherapeutic reduction of tumour. In the latter case sufficient time should be allowed for clearance of any Ig released from damaged cells. The need to swamp residual extracellular antigen with antibody must be taken into account in planning: it represents a strong argument in favour of monoclonal anti-idiotype (Miller *et al.*, 1982), which in principle at least is available in indefinitely large amount.

Appreciable killing of CW and probably of OJ cells was observed without reducing the extracellular idiotype to zero. There is a suggestion here that the effective antibody association constant might be greater for antigen on the cell surface than for antigen in the fluid phase. Uncertainties arise because there may have been transient local elimination of extracellular idiotype, and because small immune complexes might show persistent activity in assays for idiotype.

Antigenic modulation, originally defined as antibody-induced resistance to the cytotoxic action of antibody plus complement (Boyse & Old, 1969), is associated with redistribution of antigen-antibody complexes on the cell surface (Stackpole *et al.*, 1974). It does not require complete clearance of the complexes from the surface, and in the case of surface Ig can occur with a rapidity sufficient to provide some protection for cells confronted simultaneously by antibody and complement (Gordon & Stevenson, 1981). The poor performance of human complement in killing OJ, DH and MW cells *in vitro* was probably due largely to modulation competing with the complement cascade when antibody-coated cells were warmed to 37°C in the presence of serum.

The complete modulation of CW cells at the nadir after antibody infusion suggests that it was the modulation which permitted cellular survival *in vivo*. The better survival afforded in a guinea pig leukaemia by univalent anti-idiotype, which avoids modulation (Glennie & Stevenson, 1982) is also consistent with this interpretation. It should be noted that modulation can protect against cellular effectors (Griffin *et al.*, 1976; Stevenson *et al.*, 1982) as well as against complement. The occurrence of modulation in human tumour cells under attack by a variety of monoclonal antibodies has recently been reviewed by Ritz & Schlossman (1982).

Observations in this paper refer only to the killing of neoplastic cells in the vascular compartment. No diminution in size of tumour masses was observed, although the number of dead cells seen in the blood of CW soon after antibody infusion (Figure 6), which exceeds the total white cell count before the infusion, suggests that some cells in a readily accessible tissue compartment may have been killed and then released into the blood. Antibody-coated cells in the vascular compartment are exposed to high concentrations of complement components so it is likely that complement is here a major mechanism for cellular destruction. In contrast the usefulness of complement against cells in tissues is problematical, and some immunotherapeutic observations in animals and man suggest 'that it does not have an important role (Lanier *et al.*, 1980; Miller *et al.*, 1982). Although K cells, NK cells and macrophages are known to attack antibody-coated cells *in vitro* (e.g. Ojo & Wigzell, 1978; Kumagai *et al.*, 1981; Lawson & Stevenson, 1983) it is difficult to extrapolate these findings to environments *in vivo*. Lacking such precise knowledge makes it difficult to select antibody isotypes, and we have accordingly included in Table III the heading inadequate or inappropriate recruitment of effectors.

Transient exhaustion of complement or cellular effectors, in the killing of cells and clearing of complexes and debris, is a possible consequence of large antibody infusions. Apparently an exhaustion of phagocytic capacity led to a delay in clearing dead cells from the blood of CW. A similar brief appearance of circulating dead cells after antibody treatment was reported by Nadler *et al.* (1980).

It has long been appreciated that insufficient antigen density on a target cell can lead to a failure of antibody plus complement to lyse the cell (e.g. Linscott, 1970; Lesley *et al.*, 1974; Gordon *et al.*, 1982). Cell-mediated lysis (Lesley *et al.*, 1974) or cytostasis (Lawson & Stevenson, 1983) does not appear so susceptible. Results in the present study, particularly the better complement killing observed for CW cells, are consistent with surface antigen density having a significant role in determining the susceptibility of cells to killing by antibody in the vascular compartment.

There are indications that many patients treated for tumour with antibody will eventually exhibit a troublesome immune response to the foreign Ig (Miller & Levy, 1981; Miller *et al.*, 1981; Ritz *et al.*, 1981; Linch *et al.*, 1983). This has not yet been observed in any of our patients, nor apparently in other patients with B-lymphocytic neoplasms. This presumably is due chiefly to the extent of disease-associated immunosuppression present. Other factors tending to minimize the danger of an immune response would include the meticulous removal of aggregates from the infused Ig and concomitant treatment with cytotoxic drugs.

In surveying the factors tending to limit the efficacy of antibody therapy we do not see any single item looming as completely insoluble. There appears to be no good reason why antibody therapy should not be developed as a significant and safe means of disadvantaging neoplastic cells.

This work has been supported by the Medical Research Council, the Cancer Research Campaign, and Tenovus.

### References

BOYSE, E.A. & OLD, L.J. (1969). Some aspects of normal and abnormal cell surface genetics. *Ann. Rev. Genet.*, **3**, 269.

EADY, R.P., CHAPPLE, J.C., HOUGH, D.W. & STEVENSON, G.T. (1975). The specificity of a solid phase radio-immunoassay for human immunoglobulins. *J. Immunol. Meth.*, **7**, 179.

GALTON, D.A.G., GOLDMAN, J.M., WILTSHAW, E., CATOVSKY, D., HENRY, K. & GOLDENBERG, G.J. (1974). Prolymphocytic leukaemia. *Br. J. Haematol.*, **27**, 7.

GLENNIE, M.J. & STEVENSON, G.T. (1982). Univalent antibodies kill tumor cells *in vitro* and *in vivo*. *Nature*, **295**, 712.

GORDON, J., ANDERSON, V.A., ROBINSON, D.S.F. & STEVENSON, G.T. (1982). The influence of antigen density and a comparison of IgG and IgM antibodies in the anti-complementary modulation of lymphocytic surface immunoglobulin. *Scand. J. Immunol.*, **15**, 169.

GORDON, J., ROBINSON, D.S.F. & STEVENSON, G.T. (1981). Antigenic modulation of lymphocytic surface immunoglobulin yielding resistance to complement-mediated lysis. I. Characterization with syngeneic and xenogeneic complements. *Immunology*, **42**, 7.

GORDON, J. & STEVENSON, G.T. (1981). Antigenic modulation of lymphocytic surface immunoglobulin yielding resistance to complement-mediated lysis II. Relationship to redistribution of the antigen. *Immunology*, **42**, 13.

GRIFFIN, F.M., GRIFFIN, J.A. & SILVERSTEIN, S.C. (1976). Studies on the mechanism of phagocytosis. II. The interaction of macrophages with anti-immunoglobulin IgG-coated bone marrow-derived lymphocytes. *J. Exp. Med.*, **144**, 788.

HAMBLIN, T.J., ABDUL-AHAD, A.K., GORDON, J., STEVENSON, F.K. & STEVENSON, G.T. (1980). Preliminary experience in treating lymphocytic leukaemia with antibody to immunoglobulin idiotypes on the cell surfaces. *Br. J. Cancer*, **42**, 495.

HAUGHTON, G., LANIER, L.L., BABCOCK, G.F. & LYNES, M.A. (1978). Antigen-induced murine B cell lymphomas. II. Exploitation of the surface idiotype as tumor specific antigen. *J. Immunol.*, **121**, 2358.

HOUGH, D.W., EADY, R.P., HAMBLIN, T.J., STEVENSON, F.K. & STEVENSON, G.T. (1976). Anti-idiotype raised against surface immunoglobulin of human neoplastic lymphocytes. *J. Exp. Med.*, **144**, 960.

JAFFE, E.S. (1982). Hypothesis: follicular lymphomas – are they benign tumors of the lymphoid system? *UCLA Symp. Molec. Cell. Biol.*, **24**, 91.

KROLICK, K.A., ISAKSON, P.C., UHR, J.W. & VITETTA, E.S. (1979). $BCL_1$, a murine model for chronic lymphocytic leukaemia: use of the surface immunoglobulin idiotype for the detection and treatment of tumor. *Immunological Rev.*, **48**, 81.

KUMAGAI, S., STEINBERG, A.D. & GREEN, I. (1981). Antibodies to T cells in patients with systemic lupus erythematosus can induce antibody-dependent cell-mediated cytotoxicity against human T cells. *J. Clin. Invest.*, **67**, 605.

LANIER, L.L., BABCOCK, G.F., RAYBOURNE, R.B., ARNOLD, L.W., WARNER, N.L. & HAUGHTON, G. (1980). Mechanism of B cell lymphoma immunotherapy with passive xenogeneic anti-idiotype serum. *J. Immunol.*, **125**, 1730.

LAWSON, A.D.G. & STEVENSON, G.T. (1983). Macrophages induce antibody-dependent cytostasis but not lysis in guinea pig leukaemic cells. *Br. J. Cancer*, **48**, 227.

LESLEY, J., HYMAN, R. & DENNERT, G. (1974). Effect of antigen density on complement-mediated lysis, T-cell mediated killing and antigenic modulation. *J. Natl Cancer Inst.*, **53**, 1759.

LINCH, D.C., BEVERLEY, P.C.L., NEWLAND, A. & TURNBULL, A. (1983). Treatment of a low grade T cell proliferation with monoclonal antibody. *Clin. Exp. Immunol.*, **51**, 133.

LINSCOTT, W. (1970). An antigen density effect on the hemolytic efficiency of complement. *J. Immunol.*, **104**, 1307.

MILLER, R.A. & LEVY, R. (1981). Response of cutaneous T cell lymphoma to therapy with hybridoma monoclonal antibody. *Lancet*, **ii**, 226.

MILLER, R.A., MALONEY, D.G., McKILLOP, J. & LEVY, R. (1981). *In vivo* effects of murine hybridoma monoclonal antibody in a patient with T cell leukemia. *Blood*, **58**, 78.

MILLER, R.A., MALONEY, D.G., WARNKE, R. & LEVY, R. (1982). Treatment of B-cell lymphoma with monoclonal anti-idiotype antibody. *N. Engl. J. Med.*, **306**, 517.

NADLER, L.M., STASHENKO, P., HARDY, R., KAPLAN, W.D., BUTTON, L.N., KUFE, D.W., ANTMAN, K.H. & SCHLOSSMAN, S.F. (1980). Serotherapy of a patient with a monoclonal antibody directed against a human lymphoma-associated antigen. *Cancer Res.*, **40**, 3147.

NISONOFF, A. & BANGASSER, S.A. (1975). Immunological suppression of idiotypic specificities. *Transplant. Rev.*, **27**, 100.

OJO, E. & WIGZELL. H. (1978). Natural killer cells may be the only cells in normal mouse lymphoid cell populations endowed with cytolytic ability for antibody-coated tumour target cells. *Scand. J. Immunol.*, **7**, 297.

RITZ, J., PESANDO, J.M., SALLAN, S.E. & 4 others. (1981). Serotherapy of acute lymphoblastic leukaemia with monoclonal antibody. *Blood*, **58**, 141.

RITZ, J. & SCHLOSSMAN, S.F. (1982). Utilization of monoclonal antibodies in the treatment of leukemia and lymphoma. *Blood*, **59**, 1.

STACKPOLE, C.W., JACOBSON, J.B. & LARDIS, M.P. (1974). Antigenic modulation *in vitro*. I. Fate of thymus-leukemia (TL) antigen-antibody complexes following modulation of TL antigenicity from the surfaces of mouse leukemia cells and thymocytes. *J. Exp. Med.*, **140**, 939.

STEVENSON, F.K., ELLIOTT, E.V. & STEVENSON, G.T. (1977). Some effects on leukaemic B lymphocytes of antibodies to defined regions of their surface immunoglobulins. *Immunology*, **32**, 549.

STEVENSON, F.K., HAMBLIN, T.J. & STEVENSON, G.T. (1981). The nature of the immunoglobulin G on the surface of B lymphocytes in chronic lymphocytic leukemia. *J. Exp. Med.*, **154**, 1965.

STEVENSON, F.K., HAMBLIN, T.J., STEVENSON, G.T. & TUTT, A.L. (1980). Extracellular idiotypic immunoglobulin arising from human leukemic B lymphocytes. *J. Exp. Med.*, **152**, 1484.

STEVENSON, G.T., ELLIOTT, E.V. & STEVENSON, F.K. (1977). Idiotypic determinants on the surface immunoglobulin of neoplastic lymphocytes: A therapeutic target. *Fed. Proc.*, **36**, 2268.

STEVENSON, G.T., GLENNIE, M.J. & GORDON, J. (1982). The killing of lymphoma cells by univalent derivatives of tumor-specific antibody. *UCLA Symp. Molec. Cell. Biol.*, **24**, 459.

STEVENSON, G.T., SMITH, J.L. & HAMBLIN, T.J. (1983). *Immunologucal Investigation of Lymphoid Neoplasms.* Edinburgh: Churchill Livingstone.

STEVENSON, G.T. & STEVENSON, F.K. (1975). Antibody to a molecularly-defined antigen confined to a tumour cell surface. *Nature*, **254**, 714.

WERNET, P., FEIZI, T. & KUNKEL, H.G. (1972). Idiotypic determinants of immunoglobulin M detected on the surface of human lymphocytes by cytotoxicity assays. *J. Exp. Med.*, **136**, 650.